# Reactive Oxygen Species: From Tumorigenesis to Therapeutic Strategies in Cancer

**DOI:** 10.1002/cam4.70947

**Published:** 2025-05-16

**Authors:** Iqra Attique, Zahra Haider, Maha Khan, Samina Hassan, Mohamed Mohamed Soliman, Wisam Nabeel Ibrahim, Sumaira Anjum

**Affiliations:** ^1^ Department of Biotechnology Kinnaird College for Women University Lahore Pakistan; ^2^ Department of Biotechnology Lahore College for Women University Lahore Pakistan; ^3^ Department of Botany Kinnaird College for Women University Lahore Pakistan; ^4^ Clinical Laboratory Sciences Department Turabah University College, Taif University Taif Saudi Arabia; ^5^ Biochemistry Department Faculty of Veterinary Medicine, Benha University Toukh Egypt; ^6^ Department of Biomedical Science, College of Health Sciences, QU Health Qatar University Doha Qatar

**Keywords:** cancer, homeostatic dysregulation, reactive oxygen species, signal transducer, tumorigenesis

## Abstract

**Background:**

Reactive oxygen species (ROS), a class of highly reactive molecules, are closely linked to the pathogenesis of various cancers. While ROS primarily originate from normal cellular processes, external stimuli can also contribute to their production. Cancer cells typically exhibit elevated ROS levels due to disrupted redox homeostasis, characterized by an imbalance between antioxidant and oxidant species. ROS play a dual role in cancer biology: at moderate levels, they facilitate tumor progression by regulating oncogenes and tumor suppressor genes, inducing mutations, promoting proliferation, extracellular matrix remodeling, invasion, immune modulation, and angiogenesis. However, excessive ROS levels can cause cellular damage and initiate apoptosis, necroptosis, or ferroptosis.

**Methods:**

This review explores molecular targets involved in redox homeostasis dysregulation and examines the impact of ROS on the tumor microenvironment (TME). Literature from recent in vitro and in vivo studies was analyzed to assess how ROS modulation contributes to cancer development and therapy.

**Results:**

Findings indicate that ROS influence cancer progression through various pathways and cellular mechanisms. Targeting ROS synthesis or enhancing ROS accumulation in tumor cells has shown promising anticancer effects. These therapeutic strategies exhibit significant potential to impair tumor growth while also interacting with elements of the TME.

**Conclusion:**

The ROS serve as both promoters and suppressors of cancer depending on their intracellular concentration. Their complex role offers valuable opportunities for targeted cancer therapies. While challenges remain in precisely modulating ROS for therapeutic benefit, they hold promise as synergistic agents alongside conventional treatments, opening new avenues in cancer management.

## 1 Introduction

Aerobic eukaryotic organisms experience an absurdity in their existence known as the “oxygen paradox” whereby oxygen is vital for their survival, yet it may also be inherently hazardous for their sustenance [1]. An elaboration of this concept lies within the presence of reactive oxygen species (ROS), a group of reactive molecules and free and non‐free radicals that are derived from molecular oxygen and include hydroxyl radicals (OH^•^), hydrogen peroxide (H_2_O_2_), superoxide radicals (O_2_^−^), and singlet oxygen (^1^O_2_) [2, 3]. The primary source of ROS in the body is cellular metabolism, as the by‐product of the individual activity of different organelles adds up to generate the overall oxidative stress. The mitochondria, responsible for the process of oxidative phosphorylation, prove to be the main contender in generating ROS, producing approximately 90% of the total burden [4]. Moreover, NAPH oxidases, often associated with plasma membrane proteins [5], cyclooxygenases present in the cytosol [6], and peroxisomes carrying out lipid metabolism all contribute to the production of these molecules [7]. Cells may also produce ROS in response to extracellular stimuli such as hormones, xenobiotics, or the invasion of pathogens [8].

In normal cell physiology, ROS functions as intracellular signaling molecules mediating a range of biological processes such as apoptosis, immune response, cell proliferation, mitoptosis, inflammation, fibrosis, and autophagy [9]. ROS indirectly impact these processes by acting as second messengers and governing the kinases, phosphatases, and transcription factors involved in those pathways [5]. Furthermore, their importance transcends functional activities, as they are also involved in the formation of cellular structures such as protein complexes [10]. Nevertheless, aerobic organisms have evolved different mechanisms in order to maintain a balance between the production and elimination of ROS because their unregulated presence can lead to non‐specific reactions with RNA, DNA, proteins, lipids, and carbohydrates present within the cell as the radical molecules seek stability [11]. Eventually, a significantly elevated level of ROS causes irreversible cellular damage that ultimately leads to programmed cell death by necroptosis, apoptosis, or other pathways [12]. Moreover, environmental pressures such as frequent exposure to radiation and chemicals or unhealthy lifestyle choices, specifically the excessive consumption of alcohol, can naturally increase the synthesis of ROS in the body [13]. As ROS are pro‐inflammatory molecules, they overstimulate certain signal pathways that lead to the development of a range of diseases including neurodegenerative disorders such as Alzheimer's and Parkinson's [14], various autoimmune diseases [15], metabolic syndromes [16], respiratory diseases [17], and vascular disorders [18].

**FIGURE 1:**
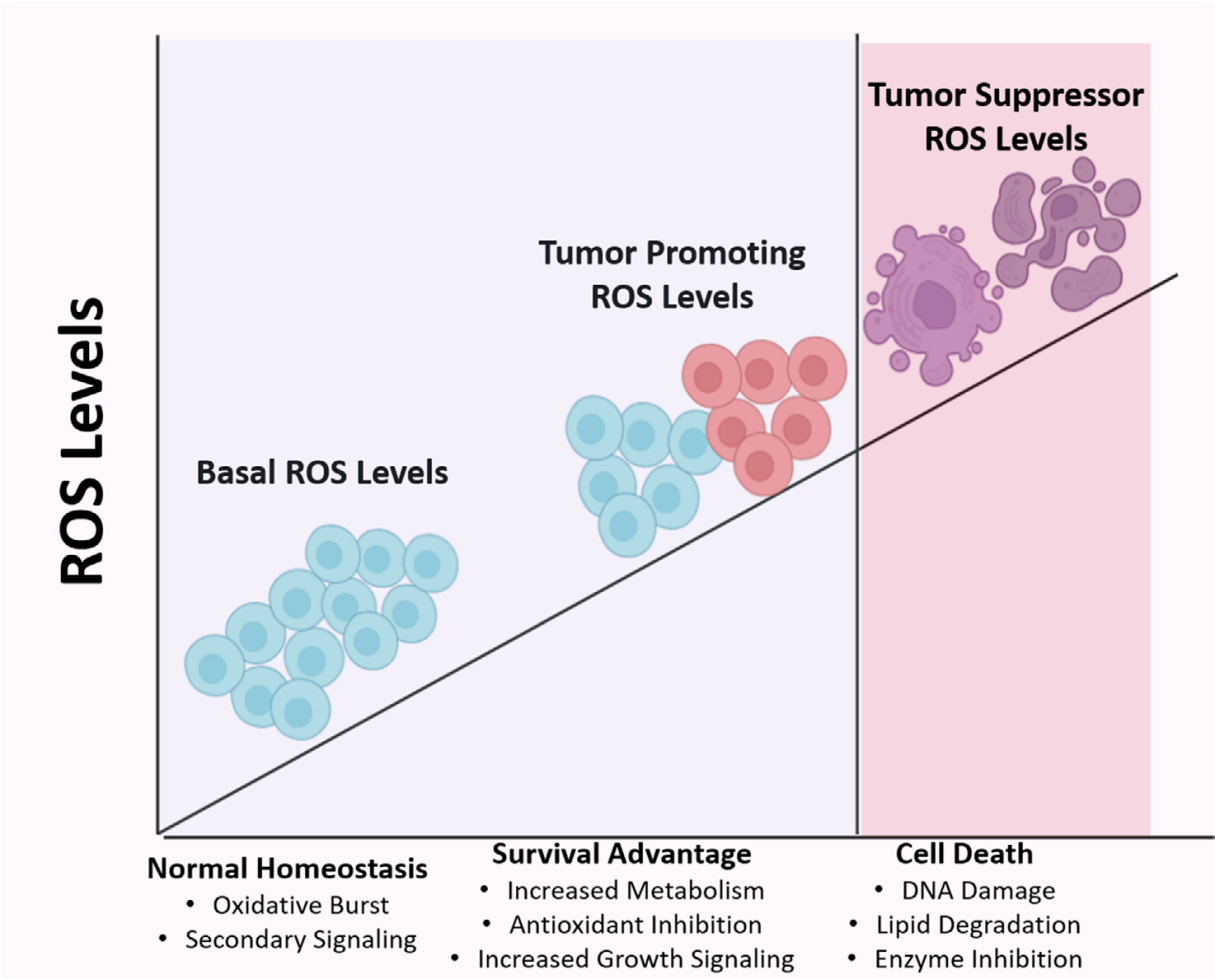
The figure illustrates the differential level of ROS in three cellular contexts‐normal, tumor‐promoting, and tumor‐suppressing conditions. In normal conditions, ROS levels are maintained at a balanced state essential for normal homeostasis. Under tumor‐promoting conditions, moderately increased ROS levels are observed along with increased metabolism, growth signaling, and antioxidant inhibition. In tumor‐suppressing conditions, elevated ROS levels promote DNA damage and cell death, mostly through apoptosis.

Cancer development is a continuously evolving phenomenon in which the initial stages mostly comprise resident host cells that are molecularly and physically altered to support the growth and progression of the tumor [19]. The cellular environment surrounding the tumor, termed the tumor microenvironment (TME), often assists cancer cell invasion, survival, dissemination, and is primarily comprised of blood vessels, immune cells, extracellular matrix, chemical modulators, microbial population, and stromal cells. However, the conformation of the TME may vary among different types of malignancies [20]. Tumor cells exhibit a significant increase in metabolic rate, relative hypoxia, and genetic mutation leading to an elevated production of ROS [21]. Nevertheless, research shows a rather paradoxical relationship between the level of ROS and cancer progression as low or moderate quantities of ROS have been reported as cancer promoters, acting as signal transducers to activate cancer cell division, invasion, tumorigenesis, migration, and drug resistance, whereas high levels of ROS damage organelles and membranes triggering programmed cell death, which proves to be harmful for the tumor, as demonstrated in Figure 1 [22]. As a result, most chemotherapeutics and radiotherapies are designed on the principle of ROS induction that increases the level of ROS above a certain threshold, yielding an anti‐cancerous effect on the targeted cells [23]. However, contemporary studies further elaborate on the counter adaptation of tumor cells to high ROS levels, which involves the activation of antioxidant pathways by regulating the transcription of pertinent genes in order to clear the radicals; thus, the development of antioxidant therapy remains under refinement [24].

The present review focuses on the various cellular targets that experience dysregulation in redox homeostasis leading to the generation of ROS, their impact on the tumor microenvironment (TME), and subsequently their bidirectional role in mediating cancer development. Furthermore, it also elaborates on the therapeutic implications of ROS in the treatment of cancer, the challenges posed during the development of such complexly targeted therapies, and the future prospects associated with them.

## 2 Sources of ROS in Tumors

### 2.1 Mitochondrial ROS

Although ROS can be generated by several exogenous influences, studies reveal the main contributor of intracellular oxidative stress to be the mitochondria (mROS), an endogenous factor [25]. Oxidative phosphorylation, the primary energy‐yielding (in the form of ATP) process of the body, involves the electron transport chain (ETC) that occurs on the inner mitochondrial membrane. Five protein complexes, embedded in the membrane, are involved in the reaction: complex I (ubiquinone oxidoreductase), complex II (succinate dehydrogenase), complex III (cytochrome c reductase), complex IV (cytochrome c oxidase), and complex V (ATP synthase), out of which complex I and III experience the largest potential energy fluctuations in electrons associated with oxygen reduction [26, 27]. The outflow of electrons interacts with molecular oxygen leading to the generation of superoxide at the flavin mononucleotide (FMN) site of complex I and the Q cycle of complex III. Complex I takes electrons from (nicotine adenine dinucleotide) NADH, passes them through a iron–sulfur clusters, and converts ubiquinone (Q) to ubiquinol (QH_2_). During this process, it pumps four protons into the intermembrane space, generating ATP and ROS [28]. Similarly, complex III is divided into two sites, the inner site (Qi), facing the matrix, and the outer site (Qo), facing the intermembrane space. During the Q cycle, an intermediate known as ubisemiquinone is autoxidized, leaking electrons to oxygen, which results in the generation of most superoxides [29]. Complex III generally produces ROS towards the intermembrane space (from Qo site) and the matrix (from Qi site) while complex I and II generate ROS only towards the matrix [29]. The superoxides are in turn converted to H_2_O_2_ by superoxide dismutase 1 (SOD1) in the intermembrane space or by the SOD2 present in the matrix [30].

Other than the ETC, certain mitochondrial enzymes are also responsible for the generation of mROS, particularly glycerol‐3‐phosphate dehydrogenase (responsible for the oxidation of glycerol‐3‐phosphate and the reduction of Q to QH_2_) that supplies electrons to the ETC, electron‐transferring flavoprotein‐ubiquinone oxidoreductase (ETF‐QOR), dihydrolipoamide dehydrogenase (subunit of ketoglutarate dehydrogenase enzyme complex), p66shc/cytochrome c, and pyruvate dehydrogenase [31, 32, 33]. The numerous metabolites, along with mROS and ATP, participate in the progression of cancer [34]. Furthermore, enzymes of the TCA cycle, including pyruvate dehydrogenase, acyl Co‐A dehydrogenase, α‐ketoglutarate dehydrogenase, and glycerol‐3‐phosphate dehydrogenase, also undergo cancer‐induced mutations resulting in unregulated ROS production [35].

Genetic mutations in the mitochondrial DNA (mtDNA) also cause an aberrant generation of ROS mainly due to the abnormal oxidative phosphorylation activity and a spike in the rate of glucose uptake and lactate formation, termed as the Warburg effect [36, 37]. Both these phenomena have been associated with tumorigenesis and cancer development [38, 39]. Mutations in the mtDNA of prostate cancer cells are associated with a decrease in the capacity of the NADH pathway and a sudden shift to the succinate pathway for energy metabolism directly impacting cancer progression [40]. Moreover, alteration in mtDNA and uncontrollable mROS can exclusively trigger different oncogenic cascades such as the TFB2M signaling pathway and EGFR signaling pathway, respectively [41, 42].

### 2.2 Oxidative Enzymes

Oxidative enzymes like NADPH oxidase, nitric oxide synthase, xanthine oxidase, and peroxisomal constituents also play an integral role in the production of ROS [43]. Among these, the NADPH oxidase (NOX) exists in five isoforms, that is, NOX1, NOX2, NOX3, NOX4, and NOX5, and is second to the mitochondria as a major source of ROS in the body [44]. The NOXs are trans‐membrane enzymes responsible for transferring electrons from the cytosol to an oxygen molecule (O_2_) on the other side of the plasma membrane in order to generate hydrogen peroxide or a superoxide [43]. In normal physiology, their activity is strictly regulated by transcription factors and other molecular approaches, thus controlling the production of ROS. However, an imbalance results in undesirable DNA damage, leading to a high rate of somatic mutations in tumor cells [45, 46]. Moreover, cancer cells, with a higher number of mitochondria, tend to maintain an increased level of NADPH that is utilized by NOXs (mainly NOX2) to metabolize fatty acids and produce an obscene quantity of ROS that in turn creates a hypoxic environment with low glucose availability, resulting in metabolically adapted, immature, oxidative neutrophils in the TME that protect the tumor from immunosurveillance [47]. Another study revealed that inhibiting the activity of NOX1 in cell lines of hepatocellular carcinoma significantly reduced angiogenesis and the induction of a pro‐tumorigenic environment [48].

### 2.3 Trace Metals

Trace metals of intrinsic and extrinsic origin, including silver (Ag), cadmium (Cd), copper (Cu), and iron (Fe) also participate in the non‐enzymatic production of ROS, contributing towards carcinogenesis. The metal ions can constitutively activate the Nrf2 transcription factor in cancer cells, downregulating the genes responsible for the antioxidant effect, thus promoting tumor growth and inferring resistance to chemotherapy [49]. Another ion frequently involved in DNA and cellular damage is iron (Fe^2+^), which utilizes the Fenton pathway to react with hydrogen peroxide and produce hydroxyl radical [50].

### 2.4 Exogenous Triggers

Lastly, multiple external factors also trigger the production of ROS in cancer cells. These exogenous factors include various types of drugs, ionizing radiation, air pollutants, certain food groups, bacteria or viruses, and cigarette smoke [51]. For instance, one puff of a cigarette releases an estimated 10^15^ radicals in the air that directly or indirectly induce oxidative damage in both passive and active smokers [52]. In addition, ionizing radiation, particularly UV and X‐rays, can oxidize water, resulting in the generation of hydroxyl radicals [53]. However, ROS produced due to exogenous triggers are often early events in cancer development, and their role in metastasis is still under debate.

### 2.5 Effect of ROS on Oncogenes and Tumor Suppressor Genes

In comparison with normal cells, cancer cells produce much higher levels of ROS mainly stimulated by oncogene activation and damage‐induced redox dysregulation. The abnormalities are related to the electron transport chain, NADPH oxidases, and centrosome yield O_2_^•‐^ ions, while an imbalance in 5‐lipoxygenase and protein folding activities of the endoplasmic reticulum generates H_2_O_2_ [54]. Some of the commonly reported oncogenes that elevate ROS include: STAT3 (alters mitochondrial metabolism and activates NOX4), BCL‐2 (targets mitochondria), Ras (alters mitochondrial functioning and activates NOX2 and NOX4), Rac1 (activates NOX1), and MYC (reduces mitochondrial volume and downregulates PGC‐1α) [55, 56, 57].

Moreover, tumor cells may also exhibit high ROS levels due to dysregulation of the expression of antioxidant enzymes due to the inactivation of tumor suppressor genes such as TP53 and post‐translational modifications of antioxidant enzymes that impart them with pro‐oxidant characteristics, the acetylation of SOD2 being a prominent example [58, 59]. In addition, immune cell– secreted TNF‐α may also induce cancer cells to produce ROS that further trigger tumor‐promoting cascades [60].

## 3 Redox Homeostasis

While ROS production assists the process of tumorigenesis, at a certain concentration it arrests the cell cycle and can induce apoptosis, senescence, or ferroptosis; thus, in order to survive the harsh conditions, cancer cells activate the transcription of antioxidant enzymes [21]. The redox regulators that govern the activity of antioxidant enzymes in cancer cells include nuclear factor erythroid‐2‐related factor 2 (*Nrf2*), ataxia telangiectasia‐mutated (*ATM*), forkhead homeobox type O family (*FoxOs*), and apurinic/apyrimidinic endonuclease 1/redox factor‐1 (*APE1 Ref‐1*). Among these, *Nrf2* proves to be the master modulator, as it regulates most of the cellular antioxidant response, and any form of dysregulation in its homeostasis can result in malignancies [22].

**FIGURE 2:**
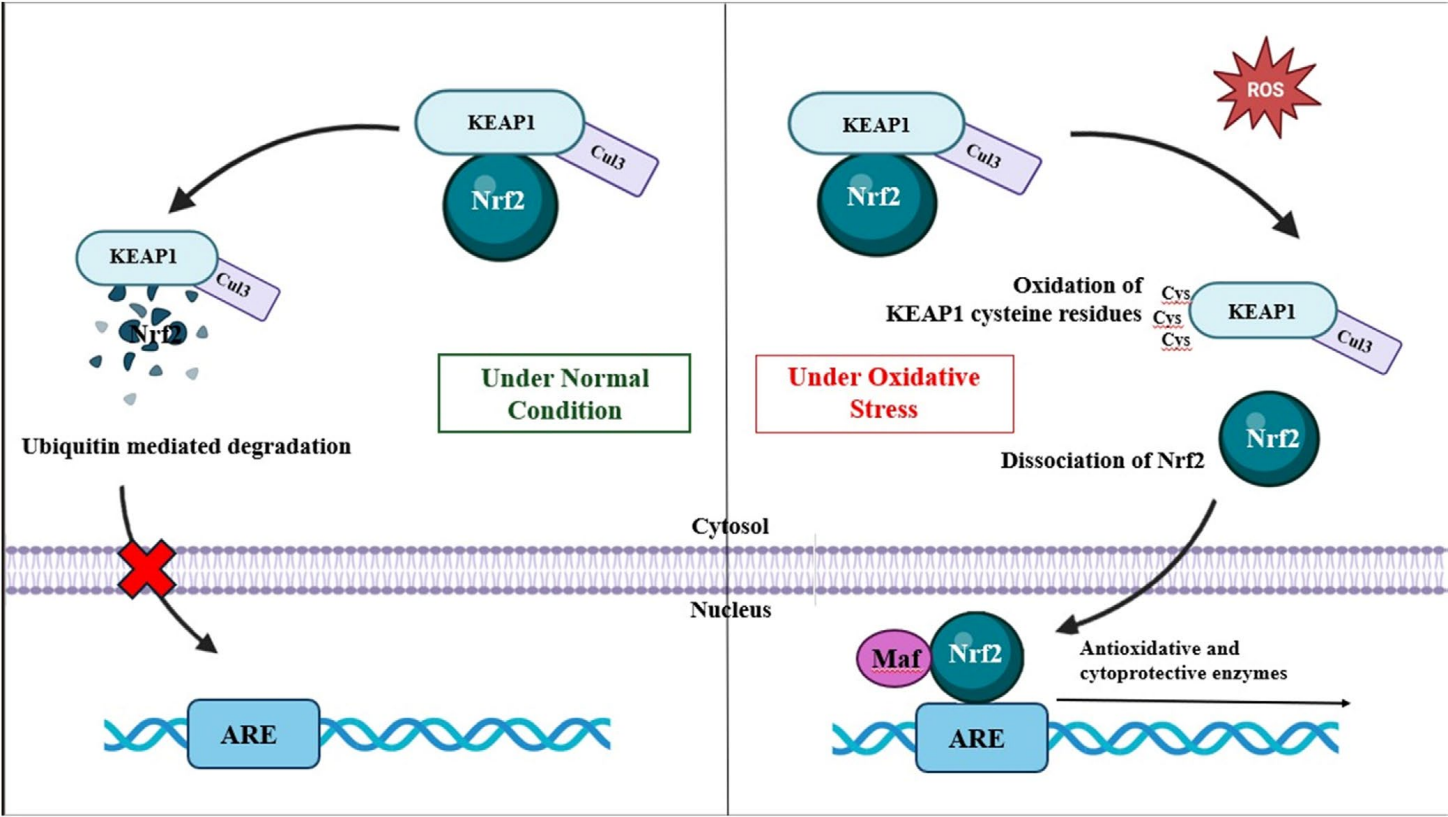
Schematic representation of the molecular mechanism of the KEAP‐Nrf2 ARE pathway: Under normal conditions, the Nrf2 remains attached to KEAP, together with Cul3, and is subjected to ubiquitination and proteasomal degradation. Under oxidative stress, the cysteine residues in KEAP oxidize, causing Nrf2 to dissociate and translocate into the nucleus where it binds to MAF proteins and induces the expression of anti‐oxidative and cytoprotective entities; ARE, antioxidant response element; Cul3, Cullin 3; KEAP1, Kelch‐like ECH‐associated protein 1; MAF, small musculoaponeurotic fibrosarcoma oncogene homolog; Nrf2, nuclear factor erythroid‐2‐related factor 2.

In the cytosol of a normal functioning cell, the activity of *Nrf2* is closely monitored by kelch‐like ECH‐associated protein 1 (*KEAP1*) as it ubiquitinates unnecessary *Nrf2* for proteosomal degradation; however, cellular oxidative stress causes Nrf2 to dissociate from *KEAP1*, and it translocates into the nucleus, binds to the antioxidant response element (ARE) of the target genes, and activates the expression of various antioxidant enzymes, as demonstrated in Figure 2 [22]. While *Nrf2* plays a cytoprotective role in normal cells, its aberrant expression in cancer cells makes it a pro‐oncogenic agent. A study conducted in 2020 by Almeida et al. revealed that breast cancer patients who displayed faster cancer progression along with lower survival also had an increased level of *Nrf2* factor, thereby forming a positive correlation between the two [61]. Further evidence was provided when *Nrf2* was knocked down in thyroid cancer cell lines, which resulted in the suppression of tumor viability and an increase in sensitivity against levatinib, a chemotherapeutic agent [62]. Likewise, an experiment involving hepatocellular carcinoma indicated a direct correlation between the upregulation of *Nrf2* and the overexpression of *Bcl‐xL* and *MMP9*, which are an anti‐apoptotic factor and metalloproteinase (factor governing the degradation of the basal lamina and aiding in cancer invasion) respectively [63]. Nevertheless, an elevated level of *Nrf2* has also been linked to tumor suppression, as reported by Shao et al., that an upregulation of *Nrf2* and GSH (glutathione) expression using curcumin leads to efficient ROS scavenging, reduction in VEGF generation, and ultimately a reduction in hepatocarcinoma aggressiveness [64]. The *KEAP1‐Nrf2* pathway can be selectively targeted for the treatment of specific cancers, as reported by Baird et al. who tested eight human cancer cell lines and stated that the DNA damaging agent mitomycin C was much more lethal to cells with abnormal *Nrf2* activation [65]. Yet another study revealed that a zinc‐curcumin (Zn(II)‐curc) compound has the potential to decrease the levels of *KEAP1* (*Nrf2* inhibitor) while increasing *Nrf2* protein and its targets, p62/SQSTM1 and heme‐oxygenase 1. This property can be exploited to enhance cancer cell sensitivity to treatments [66]. Furthermore, a drug named halofuginone, which is a low‐toxic derivative of the potent febrifugine, has the potential to induce amino acid starvation in cells, which results in the depletion of the *Nrf2* protein. Consequentially, Nrf2‐addicted cancer cells become less resistant to anti‐cancerous drugs, making treatment methods easier [67].

*FoxO* also acts as a core mediator in various oncogenic signaling pathways, controlling multiple aspects of tumor development [68]. In 2022, a study on lung cancer cells indicated that hypoxia‐inducible factor 1α (*HIF1α*) binds to the promoter region of *SCL/TAL1* interrupting locus (*STIL*), activating its expression. The *STIL* then translocates to the nucleus and associates with *FOXM1*, promoting tumor metastasis and stemness [69]. Moreover, downregulation of *FOXM1‐EZH2* signaling by the knockdown of *TRIM44* has revealed promising results in epithelial ovarian cancer cells with reduced tumor migration, invasion, and colony formation capacity [70]. Similarly, ATM is closely associated with the repair of double‐stranded DNA breaks, and research shows that inhibition of the transcription factor *ATM* in cholangiocarcinoma caused a potent response to DNA damaging agents, enhancing their cytotoxic effect, particularly in *RMCC1* and *HuCCA1* cell lines, deficient in p53 [71]. Furthermore, while analyzing the correlation of *APE2* mutations with cancer development, it was discovered that 5 of the 14 tested cancer lines displayed an abnormal increase in *APE2* expression, with a 17% overall rate of genomic alterations of *APE2* [72].

Reductases such as TXN‐1 and TXN‐2, found in the cytosol and mitochondria, respectively, also play an important role in governing the redox balance in different types of cancer cells [73]. TXNs impose an anti‐oxidant effect by reducing redox‐sensitive transcription factors, peroxiredoxins (PRDX1‐5), methionine sulfoxide reductase (Msr), and ribonucleotide reductase (RNR), and converting them into their active states [60, 74]. This process in turn leads to the oxidation of cysteine residues in the active sites of TXN‐1/2 that require subsequent reduction by selenoproteins TXNRD1 or TXNRD2 utilizing energy from NADPH and resulting in the accumulation of NADP^+^ [75]. Similar to other redox pathways, the TNX/TXNRD expression is upregulated in a variety of cancers in pursuit of countering the oxidative and hypoxic TME [76]. Lee et al. tested samples of hepatocellular carcinoma that revealed an enhanced production of TXNRD 1 which directly impacted the poor clinicopathological prognosis and patient survival [77]. Similarly, UV radiation was reported to induce skin damage through the perturbation of the TXN/TXNRD system, and a subsequent solution was sought in extracts of *Acidosasa longiligula* that positively regulated TXN1 in primary skin keratinocytes [78]. The TXN system overlaps with another antioxidant pathway involving glutathione (GSH), which serves as an important cofactor for enzymes such as glutaredoxins, glutathione peroxidases, and glutathione S‐transferases that are directly responsible for ROS scavenging [79]. The synthesis of GSH is governed by glutamate cysteine ligase (GSL) (consisting of catalytic [GCLC] and modifier [GCLM] subunits) and glutathione synthetase (GSS). Overexpression of GCLC has been associated with the poor prognosis of liver cancer patients, especially post‐surgery [80]. The resultant GSH, a tripeptide composed of glycine, glutamine, and cysteine, functions closely with the enzyme glutathione peroxidase (GPx) as a reducing agent converting peroxides such as hydrogen peroxide to alcohol and water, subsequently oxidizing GSH to glutathione disulfide (GSSG). In order to maintain an appropriate level of GSH in the cell, it is essential that the oxidized GSSG is recycled back to GSH, which is the responsibility of the enzyme glutathione reductase (GR) that utilizes NADPH during the reaction [79]. Enhanced GSH synthesis in triple negative breast cancer cells due to the activation of eIFα/ATF4 has been reported to increase radio‐resistance [81]. Redundancy between the GSH and TXN systems occurs due to GSSG, which may be reduced by enzymes involved in the TXN pathway, along with GR; thus, tumors that present with weak GSH homeostasis can still be synthetically eliminated by inhibiting the TXN/TXNRD pathway [82].

## 4 Dual Role of ROS in Cancer

The dual nature of ROS in cancer progression adds to the intricacies of the processes involved, and it is believed to be due to the diversity in their roles as secondary messengers and the fact that the manifestation of their activity depends upon their concentrations, duration, and localization. They also do not function as single molecular entities, harboring the abilities of various other molecules present within the system.

### 4.1 Tumor Promoting Effect of ROS

#### 4.1.1 ROS‐Induced Genetic Alterations

Toxic amounts of ROS manage to damage the DNA in a variety of ways, including chemical modification of bases, single‐strand breaks (SSBs), DNA crosslinking, abasic sites, double‐strand breaks (DSBs), oxidatively induced non‐DSB clustered DNA lesions (OCDLs), deamination, depurination, and depyrimidination. Some of the major products formed as a result of oxidative DNA damage include 8‐oxo‐7,8‐dihydroguanine (8‐oxoG), 8‐oxo‐7,8‐dihydroadenine (8‐oxoA), 5,6‐dihydroxy‐5,6‐dihydrothymine, and 8‐oxo‐7,8‐dihydro‐2′‐deoxyguanosine (8‐oxodG). Among these, studies have revealed 8‐oxoG to be the most reliable biomarker for oxidative stress and, in correlation, an important indicator of ROS‐induced carcinogenesis [83]. Consistent with this, when samples of patients with breast, lung, and prostate cancer were compared to those of healthy individuals, they showed a 50% higher rate of 8‐oxoG levels [84]. In addition, it is hypothesized that 8‐oxoG lesions also activate a cascade of reactions that induce mutations in adjacent DNA, particularly GC to TA transversions (common cancer genome mutation). While the DNA repair machinery of cells is always available to reverse the oxidative stress damage, ROS manage to delay the recognition of the affected DNA sites by targeting molecular intermediates involved in genome‐stability‐related pathways such as ATM, ATR, and DNA‐PKcs [85]. Moreover, ROS also impedes the activity of DNA repair enzymes by oxidizing their cysteine residues, especially OGG1, the primary enzyme involved in the excision of 8‐oxoguanine [86, 87]. Additionally, oxidative stress can lead to the formation of R loops, a non‐canonical RNA–DNA hybrid structure, that impedes molecular pathways such as transcription and replication, which further leads to genomic instability [88]. Over time, the DNA lesions accumulate with each cycle of cell division, chromosomal rearrangements occur, heterozygosity is lost, and the constant escaping of cells from the DNA repair machinery introduces many more alterations that eventually lead to permanent change and tumor transformation [89]. Thus, the role of ROS in DNA damage response (DDR) pathways is crucial in cancer development and can be exploited as a therapeutic target.

#### 4.1.2 ROS‐Mediated Cell Proliferation

Once a growing tumor reaches a diameter of 2 mm, the metabolic rate of the tumor microenvironment drastically changes and its capacity to consume oxygen exceeds the amount supplied by the blood vessels, resulting in hypoxia. Subsequently, a surge in ROS levels induces oxidative stress causing the activation or inhibition of signaling cascades involved in angiogenesis and proliferation [90]. The P13K/Akt/mTOR and the MAPK/ERK mitogenic signaling pathways, mediated by ROS, are considered to be crucial contenders in navigating these processes by activating growth factors. The tumor suppressor genes PTEN and RAS oncogene, often dysregulated in cancer cells, control the effect of ROS on the P13K/Akt/mTOR pathway. P13k signal may interact with EGFR, VEGFR, or FGFR and stimulate Akt, a proto‐oncogene, to inhibit pro‐apoptotic transcription factors and proteins. Akt further activates mTOR that regulates cell growth by phosphorylating eukaryotic translation inhibitor 4EBP1 and translation‐associated kinase S6K. Resultantly, this leads to an increase in the expression of translation initiation factor eIF4E1 that conduces protein synthesis and cell growth. Eventually, HIF‐1 α, a transcription factor that mediates oxygen homeostasis, is released that up‐regulates angiogenic genes and encourages the survival of cells in an anaerobic environment [18, 90, 91]. Yu et al. proved that suppression of the P13K/Akt/mTOR pathway led to a decrease in colon cancer cell proliferation and promoted apoptosis under the effect of Golgi phosphoprotein 3 (GOLPH3) [92]. In contrast, certain drugs influence the complex interaction between ROS and signaling pathways reversing the induction effect of ROS on P13K/Akt/mTOR to an inhibitory effect as demonstrated in a study on MCF‐7 cells that were treated with atractylodin and exhibited a significant increase in ROS that consequently blocked the P13K/Akt/mTOR pathway leading to apoptosis and autophagy [93]. Yet another drug, vernodalin, demonstrated similar results, that is, down‐regulating the FAK/P13K/Akt/mTOR and MAPK pathway in gastric cancer cells, thus inhibiting cell adhesion, proliferation, and metastasis and initiating apoptosis [94].

#### 4.1.3 ROS‐Associated Inflammation and Tumorigenesis

While each individual component of the tumor microenvironment contributes to oxidative stress, the mechanism of each cell type remains undiscovered. Studies have suggested that chronic inflammation, along with myeloid cell‐mediated ROS and tumor necrosis factor α (TNF‐α) signaling, can lead to invasive cancer [95]. Similarly, cancer‐associated fibroblasts (CAFs), a class of non‐cancerous cells that interact with the tumor, engage in a two‐way cross‐talk with ROS such that ROS converts fibroblasts into its active counterpart (CAFs) by upregulating HIF1α, and simultaneously, CAFs are also pivotal in increasing ROS concentration in cancer cells. CAFs, in turn, support carcinogenesis and metastasis, continuing the cycle of ROS production [96].

#### 4.1.4 Role of ROS in Telomere Maintenance Mechanisms

Telomeres are protective sequences found at the ends of chromosomes that shorten with each cycle of cell division and are sensitive to oxidative stress. Telomere shortening, ameliorated by the enzyme telomerase, is the core mechanism involved in aging, which is in turn associated with mitochondrial dysfunction leading to elevated ROS production [97]. Rapid cell division, a hallmark of most cancer cells, is maintained by upregulating telomerases or alternative lengthening of telomeres (ALT) through homology‐directed DNA repair [98]. As mentioned earlier, 8‐oxoG, a common DNA lesion found in cancer cells, often accumulates at telomeric ends and directly impacts the activity of telomerases, thus inducing replicative stress and telomere losses. Consequentially, the losses cause genome instability through chromosome fusion, formation of chromatin bridges, and micronuclei [99]. Furthermore, studies on the effect of telomeric 8‐oxoG on the ALT pathway revealed a paradoxical role, as the damage may help or hinder the ALT process, depending upon the phase of the cell cycle and the type of cancer cell. Corroborating this, a recent study by Thosar et al. revealed that targeting 8‐oxoG formation in telomeres activated homologous recombination and the ALT pathway, particularly in ALT cancer cells. Cancer cells that were dependent upon the ALT pathway experienced more replication stress and were also more sensitive to the 8‐oxoG telomeric damage than those using telomerases. Furthermore, it was also found that if the damage occurs during the G2 phase of the cell cycle, ALT activity is blocked and telomere repair is hindered [100]. Yet another study highlighted that R‐loop formation, induced by high ROS levels in ATRX‐deficient glioma cells, when attempted to be removed by the RNase H1 enzyme, caused the ALT process to halt, suggesting that R‐loops are essential in the activation of the ALT pathway. In addition, it was found that long‐term elevated levels of ROS can overactivate the ALT pathway, causing severe DNA damage and eventually cell death [101].

### 4.2 ROS‐Induced Tumor Suppression and Cell Death

As stated earlier, ROS mediate a variety of signaling cascades that promote cancer progression; however, when the accumulation of ROS exceeds the threshold value, their pro‐oncogenic role is modified to a tumor suppressive one, mainly by the induction of cell death processes, namely, apoptosis, necrosis, and ferroptosis [102].

#### 4.2.1 Apoptosis

Apoptosis, also referred to as type I programmed cell death, is mediated by certain cysteine proteases termed as caspases and can be initiated by one of the two core pathways: intrinsic pathway or extrinsic pathway.

The intrinsic pathway originates in the mitochondria and is dependent upon the release of proapoptotic factor cytochrome c (Cyt‐c) triggered by the interaction of the mitochondrial membrane with Bax/Bak. Normally, Cyt‐c is immobilized within the mitochondria by cardiolipin sequestration. Upon oxidation by ROS, cardiolipin dissociates from Cyt‐c and allows it to travel to the cytosol. Thereafter, Cyt‐c interacts with apoptotic protease activating factor 1 (APAF‐1) and procaspase‐9 to form a multi‐protein complex known as the apoptosome. Subsequently, the procaspase‐9 signaling cascade is initiated that, along with effector caspases–3, –6, and –7, leads to the demolition of the cell [103, 104]. Research highlighting the correlation between endogenous elements and cancer progression through the intrinsic apoptotic pathway has been reported extensively, such as in a study conducted in 2021 that revealed that transfecting MCF7 breast cancer cells with the lrg1 gene, encoding the LRG1 protein, protected the cancer cell line against apoptosis. The excessive intracellular LRG1 captured Cyt‐c, inhibiting its interaction with Apaf‐1 and preventing the onset of the intrinsic apoptosis pathway [105]. Furthermore, toxic levels of ROS also increase the permeabilization of the mitochondrial outer membrane through Bcl‐2 regulation that causes Cyt‐c to enter the cytosol and initiate the cycle [106].

The extrinsic pathway is activated by the interaction between ligands, FasL, TRAIL‐R1/2, and TNF, with the extracellular domains of their respective transmembrane receptors FasR and TNFR. The ligand‐receptor interaction triggers a cascade of events involving the reciprocation of adaptor proteins (FADD and TRADD respectively), procaspase‐8 and ‐10, and RIP1 to form the death‐inducing signaling complex (DISC) that in turn instigates apoptosis. The role of ROS in mediating this pathway is as an accelerator of the degradation of cellular FLICE‐inhibitory protein (c‐FLIP), an anti‐apoptotic factor [107]. In addition, the caspases −8 and −10 link the extrinsic pathway to the intrinsic, mitochondrial‐dependent pathway by cleaving Bid, a pro‐apoptotic protein, into truncated Bid (tBid) that subsequently blocks the activity of Bcl‐XL and Bcl‐2 in the mitochondria and instigates Bak and Bax [108].

Another route for the activation of apoptosis by elevated levels of ROS involves the activation of mitogen‐activated protein kinase (MAPK), particularly the initiation of the c‐Jun N‐terminal kinase (JNK) signaling pathway [109]. Research indicates the contribution of JNK phosphorylation in inducing apoptosis by the significant suppression of cancer progression in hepatocellular carcinoma BEL‐7402 and HepG2 cells via the activation of the ROS‐JNK‐P53 pathway [110]. Similar results were observed in intrahepatic cholangiocarcinoma cells by the proteasome inhibitor MLN2238 [111]. Moreover, the drug Curaxin CBL0137, a regulator of p53 and NF‐ĸB, was also reported as a potent inhibitor of B‐cell non‐Hodgkin's lymphoma tumor growth, and it functioned by triggering ROS generation that led to the activation of the P13K/Akt/mTOR and MAPK signaling pathways, eventually causing cell apoptosis and autophagy [112].

#### 4.2.2 Necroptosis

Necroptosis, also known as type III programmed cell death, is also driven by the interaction of a ligand and receptor, similar to the initiation of the extrinsic apoptotic pathway. The three membrane‐embedded receptors that are common between the two pathways include: death receptor 4 and 5 (DR4/5), TNFR1, and Fas [113]. However, unlike apoptosis, necroptosis is caspase‐independent and involves RIP3 in the formation of DISCIIb [114]. Mitochondrial ROS promotes necroptosis through the direct activation of the RIP1/RIP3 complex [115]. The autophosphorylation of RIPK1 triggers a cascade that results in the subsequent recruitment and autophosphorylation of RIPK3 and (mixed lineage kinase domain like protein) MLKL, respectively, leading to the formation of a functional necrosome. Finally, MLKL oligomerizes and translocates to the membrane to form membrane‐disrupting pores, rendering the dysfunctional [116]. With the aim of exploiting this pathway for the treatment of cervical cancer, a recent study treated CaSki cell lines with RETRA and concluded that it hyperpolarized the mitochondria and caused the accumulation of ROS that led to the phosphorylation of RIPK1, RIPK3, and MLKL (necroptosis) irrespective of the status of p53 [117]. Necrosis, exclusively and in conjunction with inflammatory responses, has shown evidence as a mediator of cancer prognosis [114, 118]. A 2020 research explored the anti‐tumor effect of Taraxastane on human cervical cancer cells DoTc2 and revealed remarkable anti‐proliferative properties, owing to the activation of ROS‐mediated necrosis through modulation of the JNK/MAPK signaling cascade [119]. Similarly, the growth of liver cancer cells decreased after treatment with a hybrid protein NTP‐217 that damaged the mitochondrial membrane, causing its contents to leak and inducing toxic quantities of ROS production [120].

ROS synthesis via aerobic respiration and the citric acid cycle in the mitochondria can also be induced by (i) upregulating the expression of glycogen phosphorylase (PYGL) and/or pyruvate dehydrogenase (PDH) and (ii) increasing the quantity of glutaminolysis by over‐expressing glutamate dehydrogenase‐1 and glutamate‐amino ligase [102].

#### 4.2.3 Ferroptosis

Ferroptosis is another type of regulated cell death that differs molecularly from apoptosis and necrosis with respect to its dependency on both iron and ROS to initiate. In addition, it is also characterized by the accumulation of peroxidated polyunsaturated fatty acids prior to cell death. The oxidation of phospholipids involves the Fenton oxidation cascade, which includes a series of reactions between H_2_O_2_ and labile iron to produce oxygen‐centered radicals that facilitate ferroptosis, particularly upon iron overload [121]. Furthermore, an accumulation of ROS drives autophagy‐dependent ferroptosis that causes the polarization of pro‐tumorigenic M2 macrophages and results in the formation of tumor‐associated macrophages (TAM) that subsequently promote tumor progression [122]. Contemporary research has indicated the importance of ferroptosis, induced by small molecular elicitors, in increasing the sensitivity of cancer cells to chemotherapeutics and controlling tumor metastasis and growth [123]. Gastric cancer proliferation was seen to be repressed with the treatment of Baicalin and 5‐Fu as a consequence of a surge in intracellular ROS. RNA‐seq analysis of the treated cells indicated an enriched expression of four ferroptosis‐regulating genes by ROS [124]. Likewise, a study published in 2021 treated non‐small cell lung cancer with artemisinin derivatives ART and DHA that upregulated transferrin receptor (TFRC) and downregulated cystine/glutamate transporter (xCT) which are positive and negative mediators of ferroptosis, respectively [125].

#### 4.2.4 Autophagy

Autophagy, a natural and conserved process, aims to reuse the damaged, dysfunctional components of the body through a lysosome‐dependent manner of degradation. With reference to cancer cells, autophagy regulates its activity based on context, that is, it may suppress or promote the growth of the tumor depending upon the stage of cancer and the type of tumor [126]. During the early stages of tumorigenesis, autophagy sequesters and eliminates cellular components damaged as a result of ROS attack, inhibiting the growth of the tumor. Various types of tumor present a defective autophagy pathway that conduces to a tumor suppressive environment. A study reported that Bruceine D targets the ROS/MAPK signaling pathway and leads to the induction of apoptosis and autophagy in lung cancer cells [127]. Similarly, gastric cancer development was subdued by BDH2 by triggering ROS‐mediated autophagy by promoting ubiquitination through the Nrf2 pathway [128]. However, with the presence of cancer‐associated fibroblasts (CAFs) in the TME, ROS (via autophagy) begin functioning as a communicator that enhances cross‐talk between CAFs and tumor cells, leading to cancer and metastasis promotion [129]. Moreover, autophagy also eliminates ROS‐mediated stress during tumor growth and provides additional nutrients to the cancer cells, particularly by activating autophagy in stromal cells. A study compared the CAFs isolated from ovarian cancer cells and normal fibroblasts, obtained from benign tissues, revealing that CAFs are significantly more resistant to oxidative stress, presumably through autophagy [130]. Likewise, a recent experiment on a breast cancer xenograft model revealed that elevated mitochondrial ROS in stromal cells affected HIF‐1α expression, thus inducing autophagy and enhancing tumorigenicity [131].

## 5 Impact of ROS on Tumor Microenvironment

**FIGURE 3:**
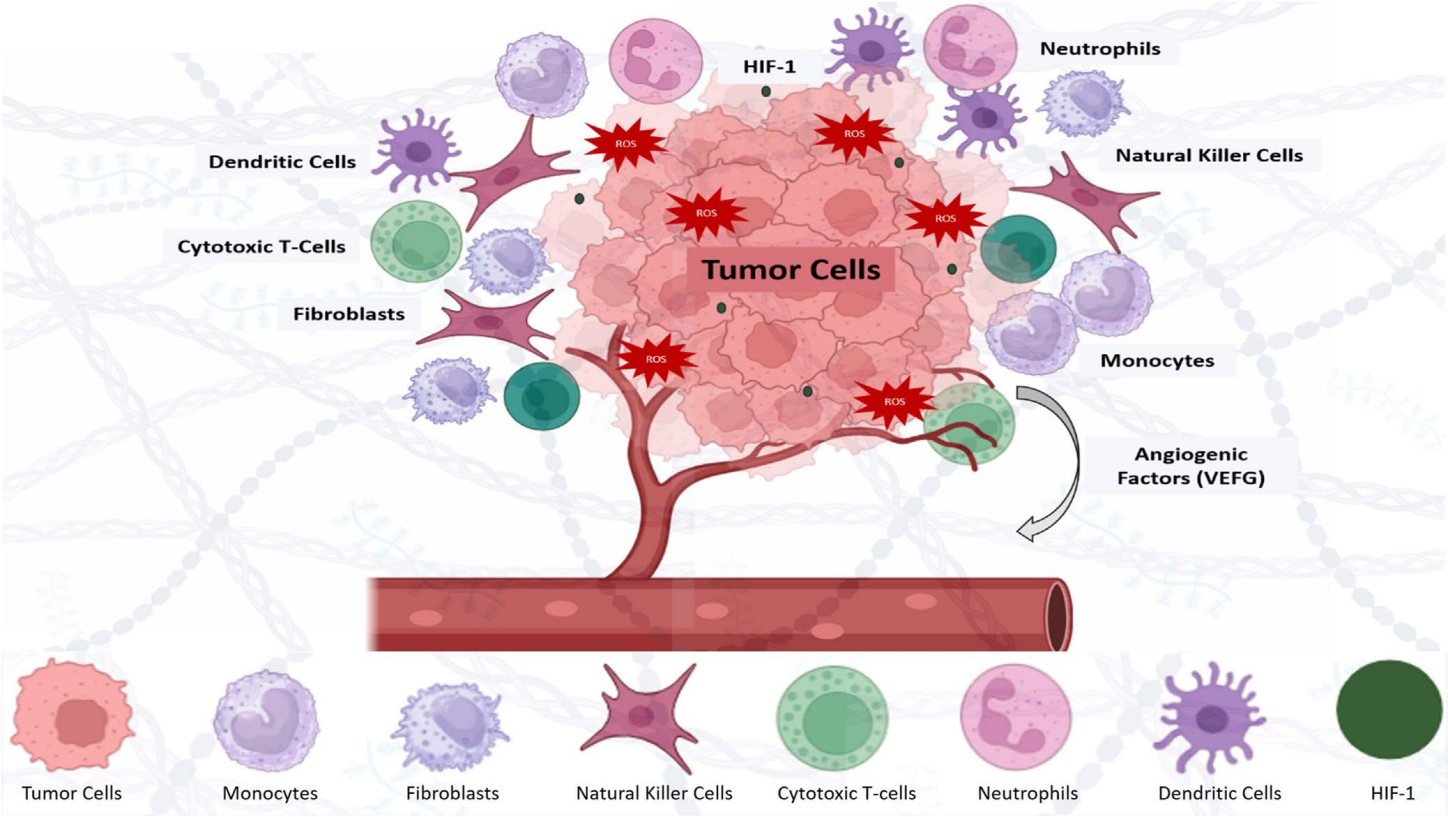
The dynamic and complex tumor microenvironment includes different cellular components such as cancer cells, immune cells, CAFs, and endothelial cells, along with non‐cellular elements such as the ECM.

The “seed and soil” hypothesis proposed by Stephen Paget in 1889 is a well‐recognized concept that deciphers the mechanism involved in the metastatic spread and progression of tumor cells [132]. It highlights the importance of the tumor microenvironment in providing an environment conducive to the survival, proliferation, and dissemination of metastatic cancer cells. TME consists of a complex and diverse plethora of different components including tumors, tumor blood vessels, stromal cells, extracellular matrix, inflammatory and immune cells, chemical cytokines, growth factors, and microbial communities as shown in Figure 3 [133, 134].

The interaction of the tumor cells and neighboring stromal cells incorporated into the dense three‐dimensional extracellular matrix (ECM) components plays a substantial role in determining the growth and spread of tumor cells [135]. Certain cells in TME are involved in facilitating tumor growth by supplying oxygen and nutrients, releasing growth factors and cytokines, and inhibiting immune responses against tumor cells. However, on the other hand, there are some elements in TME capable of tumor suppression such as cytotoxic T‐cells and natural killer cells that possess the ability to eliminate tumor cells through different mechanisms.

One of the hallmark features of malignancy is desmoplasia, which is the rapid growth of fibrous and connective tissues [136]. The fibrous tissues exhibit excessive mechanical forces on the lymphatic and blood vessels, thereby restricting the oxygen supply and creating a low oxygen or hypoxic environment. A weakly acidic environment promotes the dissemination of the disease from the primary tissue sites via different pathways and increases the production of mitochondrial ROS. Elevated ROS levels stimulate the conversion of fibroblasts to myofibroblasts, which is associated with tumor progression and metastasis [137, 138].

**FIGURE 4:**
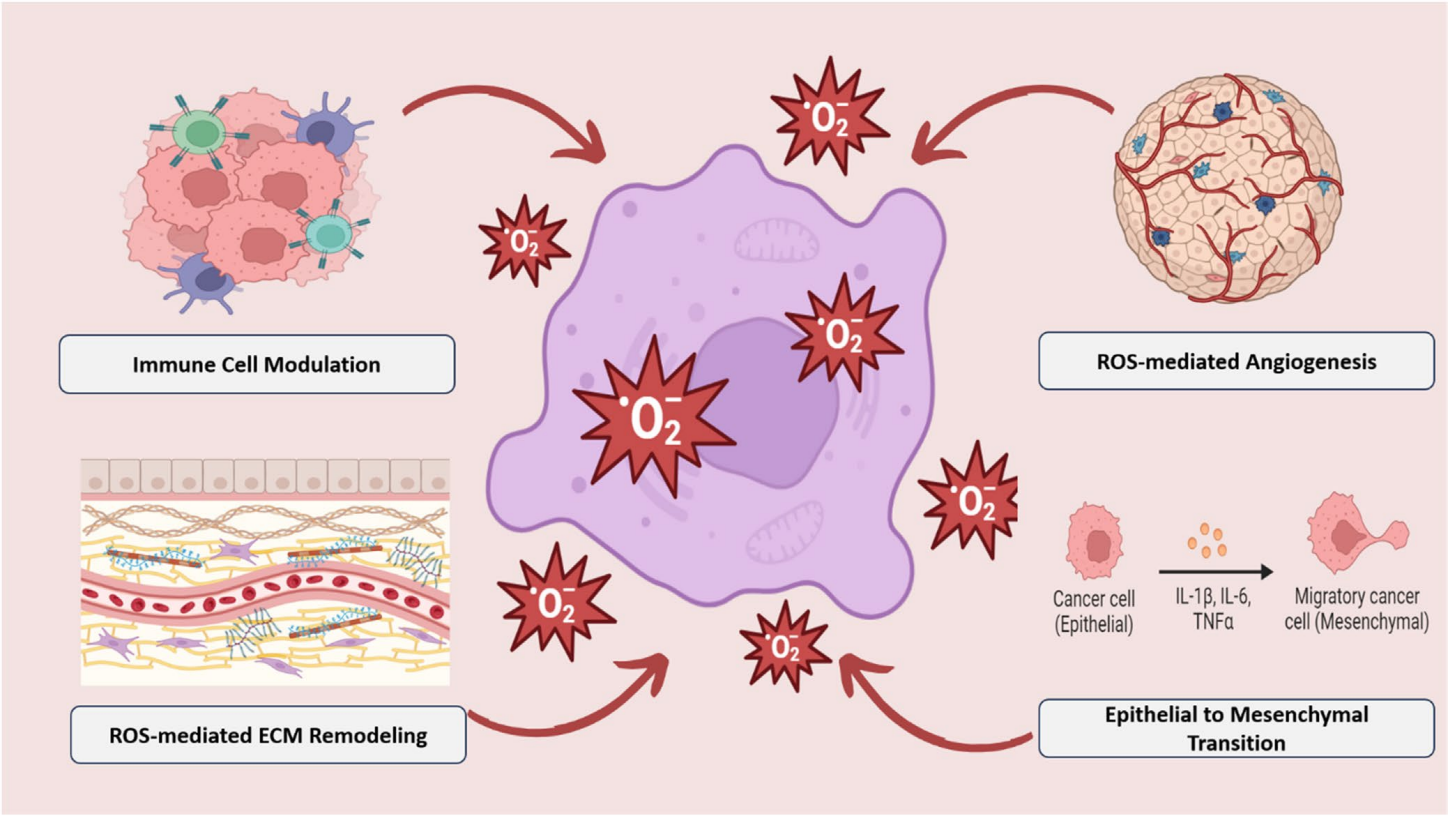
Impact of ROS on tumor microenvironment; (i) immune cell modulation; (ii) ROS‐mediated ECM remodeling; (iii) ROS‐mediated angiogenesis; (iv) epithelial to mesenchymal transition (EMT).

ROS plays a critical role in the TME by influencing angiogenesis, epithelial to mesenchymal transition (EMT), extracellular matrix (ECM) remodeling, and immune cell reprogramming as shown in Figure 4. Elevated ROS levels stimulate the secretion of angiogenic factors like VEGF, facilitating tumor vascularization and progression. ROS‐induced hypoxia in the TME activates HIF‐1α, promoting endothelial cell proliferation necessary for new blood vessel formation. Additionally, ROS triggers EMT by inducing transcription factors such as SNAIL, supporting tumor invasion and metastasis. ROS also modulates ECM remodeling by activating proteolytic enzymes and transforming fibroblasts into cancer‐associated fibroblasts (CAFs), which secrete growth factors and cytokines that enhance tumor growth and metastasis. Furthermore, ROS reprograms immune cells, either promoting tumor immune evasion by suppressing cytotoxic T‐cells and NK cells or facilitating anti‐tumor responses by activating immune pathways. Studies have shown that manipulating ROS levels can impact immune cell function, highlighting its complex role in cancer development and progression. In the following sections, each of these changes in the TME will be further elucidated.

### 5.1 ROS‐Mediated Angiogenesis

ROS has a complex and significant role in TME, modulating multiple aspects of tumor progression and host immune responses. It can drive angiogenesis by stimulating the secretion of angiogenic factors through the activation of various growth factors and cytokines in TME, thus promoting tumorigenesis and tumor progression [139]. Elevated ROS can upregulate the expression of pro‐angiogenic factors such as vascular endothelial growth factor (VEGF), which promotes tumor vascularization [139]. This positive interrelationship between the ROS levels and angiogenesis is significant in various phases of tumor development, including vascularization and dissemination.

Endothelial cell proliferation and migration are of utmost importance for forming new blood vessels. The enhanced vascularization is significant in facilitating the metastasis of cancer cells and providing oxygen and nutrition to the surrounding tumor cells [140]. In this process, hypoxia inducible factor (HIF), which is highly expressed in the hypoxic TME, assists in the activation of VEGF, which binds to its receptors, ultimately triggering different signaling pathways, including the ERK/MAPK signaling pathway [127]. This strongly facilitates the proliferation of endothelial cells, which is essential for angiogenesis. Consequently, the expression of proangiogenic factors such as VEGF and PDGF is considerably increased in tumor cells [141]. For instance, HIF‐1α stimulates angiogenesis and vascularization in hepatocellular carcinoma tumors by the stimulation of several target genes such as VEGFA, VEGFR1, and EphA1 [142]. In a study, the inhibition of CDK5 (a protein that binds to HIF‐1α) can impair the ability of HIF‐1α to activate the transcription of target genes, thereby suppressing angiogenesis [142]. Moreover, ROS production in the TME caused by both tumor and stromal cells facilitates the manifestation of cancer hallmarks by modulating different signaling cascades.

ROS produces and upregulates the expression of oncogenic factors which promote metastatic progression [143]. Various studies have substantiated a positive correlation between the levels of ROS and angiogenesis. Both endothelial and smooth muscle cells express VEGF when they are exposed to hydrogen peroxide, subsequently promoting tumor angiogenesis. A study conducted by Kim et al. demonstrated the role of a signaling protein, pS36‐p66Shc, in increasing the mtROS that in turn activates the signaling of the VEGF2 receptor. This positive feedback loop in turn promotes angiogenic responses in endothelial cells [144]. Likewise, ROS production stimulates the overexpression of NOX‐1, as a consequence of which VEGF expression in DU‐145 prostate cancer cells and NIH3T3 fibroblasts increases [145]. ROS levels orchestrate the process by which VEGF stimulates the dimerization and auto‐phosphorylation of its receptor VEGFR2, essential for the subsequent process of angiogenesis [146, 147]. Additionally, ROS plays a crucial role in sustaining the viability of cancer cells by promoting the production of NF‐kB, VEGF, and MMPs by different signaling pathways at moderate levels [148]. A study revealed that an enhanced level of intracellular ROS is involved in tumor migration by the regulation of MMP1 under hypoxic conditions [149].

### 5.2 Epithelial to Mesenchymal Transition (EMT)

Epithelial to mesenchymal transition (EMT) is a process in which epithelial cells lose their polarity and intercellular connections and are subsequently converted into mesenchymal cells. This transition essentially facilitates different phases of cancer development, including tumor growth, invasion, vascularization, and metastasis [150]. During this process, epithelial cells undergo significant cytoskeletal remodeling to support tumor metastasis. Inflammatory cells and oxidative stress due to elevated ROS levels are regarded as potent inducers of EMT in the tumor microenvironment [151].

The process of EMT is primarily driven by various transcription factors (EMT‐TFs), including SNAIL, TWIST, and ZEB families [152]. ROS is known to mediate EMT, consequently assisting in the formation of a tumorigenic microenvironment. ROS can induce the expression of SNAIL. However, another study reported the overexpression of SNAIL in enhancing the ROS levels, both potentially leading to stimulation of EMT [153, 154].

### 5.3 ECM Remodeling

ECM not only provides physical support and integrity to the cells but also plays an important role in different biological processes such as cell survival and differentiation, and contributes to the pathogenesis of various diseases including cancer. It is composed of a complex network of different elements including proteoglycans, hyaluronic acid, and fibrous proteins such as fibronectin, laminin, collagen, and elastin [155]. The biochemical and biomechanical characteristics of ECM in tumor cells are substantially altered to provide a structural and dynamic framework to tumor cells thereby supporting their growth and development. For instance, tumor cells are reported to contain dense, modified ECM that contributes 60% of the tumor mass [12]. Moreover, in normal cells, collagen provides tensile strength and stability and facilitates cell migration and adhesion; however, in cancerous cells, there is a noticeable increase in crosslinking and deposition of collagen essential to tumor metastasis.

During cancer development and metastatic spread, tumor cells undergo active changes in their interactions with ECM components, growth factors, and ECM‐associated cytokines as well as the stromal cells [156]. Cancer advancement is promoted by the synergistic action of these cells, which assist in ECM remodeling. For metastatic invasion, the tumor cells must first identify, adhere to, and disrupt the basement membrane with the help of proteolytic enzymes. The major groups of proteolytic enzymes involved in tumor progression include (i) matrix metalloproteinases (MMP), (ii) serine proteases, and (iii) cysteine proteases. ROS modulates the expression and activity of these proteolytic enzymes by utilizing different intracellular pathways [157]. This leads to extensive ECM remodeling, creating conditions conducive to cancer cell migration. For instance, ROS increases the expression of MMP‐2 and MMP‐9 in fibrosarcoma cells, promoting the ECM breakdown, angiogenesis, release of growth factors such as cytokines, and inhibition of T‐cell proliferation and function that allow tumor cells to metastasize at distant locations [158].

Moreover, activated fibroblasts known as CAFs are essentially involved in ECM remodeling. They promote cancer progression by secreting growth factors and cytokines and by stimulating angiogenesis. Fibroblasts and myofibroblasts are two major categories of CAFs. Secreting factors such as CXCL12 stimulate various signaling pathways such as AKTs responsible for promoting tumor development [159].

Accumulating studies highlight the pivotal role of ROS in driving myofibroblast differentiation, which further remodels the ECM to support tumor growth and metastasis. Huang and his colleagues demonstrated that oxidative stress generated by ROS in the extracellular tumor microenvironment resulted in the trans‐differentiation of human monocytes into CAFs, consequently inhibiting the immune responses and promoting fibrosis and tumor dissemination [160]. Similarly, in another study, TGF‐β and SDF‐1 signaling pathways promoted the differentiation of fibroblasts into myofibroblasts in a ROS‐dependent manner, thus supporting substantial tumor growth progression. Additionally, an increase in the expression of antioxidant enzymes such as GSH and TXR inhibited myofibroblast differentiation in prostate cancer due to decreased ROS levels [161].

### 5.4 ROS Reprogramming of Immune Cells

ROS can modulate immune responses by altering the activity and behavior of different immune cells. The double‐edged sword role of ROS can either promote tumor immune evasion by suppressing the activity of cytotoxic T‐cells and natural killer cells or facilitate an anti‐tumorigenic response by stimulating signaling pathways to recruit and activate immune cells [102, 162].

#### 5.4.1 T‐Cells

The presence of ROS in TME significantly determines the activation and functioning of different subsets of T‐cells. Low concentrations of ROS are required for T‐cell activation, differentiation, and proliferation, whereas elevated concentrations of ROS can lead to immunosuppression along with an increase in T‐cell apoptosis within the TME [163]. This is because of the induction of oxidative stress, which impairs the normal immunological function of T‐cells, leading to T‐cell hypo‐responsiveness in cancer‐affected individuals. Ligtenberg and colleagues, in a study, engineered T‐cells co‐expressing CAR and CAT receptors to target cancer cells. The engineered T‐cells (CAR‐CAT) exhibited enhanced anti‐oxidant activity by reducing the accumulation of ROS in tumor cells. The reduction in oxidative stress allowed the T‐cells to eliminate the cancer cells effectively [164].

#### 5.4.2 Natural Killer Cells

Moreover, oxidative stress due to the accumulation of ROS in the TME can suppress the normal functioning of natural killer (NK) cells. For instance, an increased concentration of hydrogen peroxide in the TME stimulates NK cell death, eventually limiting NK cell infiltration. It also adversely influences the cytotoxic ability of T cells and NK cells, hence hindering their ability to target and eliminate cancer cells effectively [165]. A study conducted by Mellqvist highlights the impact of ROS on NK cells functioning in chronic myelogenous leukemia (CML). The paracrine production of ROS impaired the normal functioning of NK cells, and they were subsequently subjected to apoptosis due to oxidative stress. ROS scavengers such as catalase and histamine, however, restored the cytotoxic ability of NK cells [166].

#### 5.4.3 Dendritic Cells (DCs)

Dendritic cells (DC) are an important component of the immune system. They are majorly responsible for presenting antigens to T‐cells, eventually eliciting an appropriate anti‐tumor immune response [167, 168]. Studies confirm that ROS has a complex influence on DCs, involving alterations in metabolic pathways and gene expression. High levels of ROS can have detrimental effects on DCs. This could be explained by the excessive oxidative stress and chronic ER stress responses that hinder the ability of DCs to process and present antigens to T‐cells [169, 170].

#### 5.4.4 Tumor‐Associated Macrophages (TAMs)

Tumor‐associated macrophages (TAMs) account for a significant portion of tumor‐infiltrating immune cells in TME. They are widely classified into two major subtypes: classical activated M1 and alternatively activated M2‐type macrophages, typically associated with anti‐tumor and pro‐tumor effects respectively [171, 172]. These two subtypes can switch into one another in response to different stimuli. This adaptability is referred to as Macrophage plasticity [173]. ROS‐induced NF‐κB signaling pathway activation promotes the immunosuppressive phenotype of macrophages. This shift is marked by an increase in the transcription and production of PD‐L1, which consequently inactivates cytotoxic T‐cells. Moreover, NF‐κB activation also enhances the release of chemokines by macrophages, which create an environment that promotes tumor invasion and progression [174]. Similarly, in another study, NOX4 expression in cancer cells resulted in the elevated production of ROS, which subsequently stimulated the release of various cytokines through the PI3K/Akt pathway. The released cytokines played a notable role in M2 polarization and macrophage migration. M2 macrophages exhibited increased JNK activity and released HB‐EGF, which promoted the proliferation of cancer cells [175]. On the other hand, a study demonstrated that the elevated levels of ROS facilitate the activation of ATM kinase, which drives the differentiation of macrophages into the pro‐inflammatory M1 phenotype [176]. The role of ROS in stimulating M1 polarization has also been supported by various studies [177, 178].

#### 5.4.5 Neutrophils

Neutrophils constitute a major portion of the immune system, primarily involved in the elimination of infection during an immune response through multiple mechanisms. They are involved in tumor invasion, progression, and angiogenesis [179]. However, the role of tumor‐associated neutrophils (TANs) in altering the tumor microenvironment is still debated, with several studies demonstrating both pro‐and anti‐tumor effects [180, 181]. Bekes et al. reported the elevated production of MMP9 in the tumor microenvironment by tumor‐associated neutrophils, which subsequently contribute to tumor progression, invasion, and neovascularization in the mouse transplantation models [182]. Similarly, the neutrophils‐released ROS in the tumor microenvironment supported tumor growth by causing oxidative stress and generating genetic mutations [183]. In contrast, Gershkovitz et al. reported that the interaction of neutrophils with cancer cells resulted in the release of hydrogen peroxide, which activated the TRMP2 channel, leading to a lethal influx of Ca^2+^ ions. This influx initiated a cascade of events that ultimately led to tumor cell apoptosis [184].

#### 5.4.6 Regulatory T‐Cells (Tregs)

Regulatory T‐cells (Tregs) exhibit potent immunosuppressive effects in the TME by maintaining a supportive environment that favors tumor growth. It facilitates tumorigenesis and tumor survival and suppresses anti‐tumor immunological pathways [185]. Tregs are significantly involved in tumor immune escape, which is a phenomenon by which tumor cells grow, invade, and spread to different regions by evading immunological barriers [186, 187]. Tregs hinder antigen presentation by DCs, disrupt the function of CD4+ helper cells, and release CTLs, contributing mutually to the suppression of anti‐tumor immunity [188]. This is due to the release of various cytokines such as IL‐10, IL‐35, and TGF‐β. Additionally, various subsets of T‐cells present in the TME express IL‐10 and IL‐35, notably involved in T‐cell exhaustion in tumor cells. The release of granzyme and perforin to induce apoptosis and cAMP to disrupt the metabolic functioning of neighboring cells is another strategy adopted by Tregs to eliminate the target cells directly [189]. Maj et al. reported an increase in the immunosuppressive effect of Tregs in the presence of high levels of ROS and oxidative stress in the tumor microenvironment, potentially facilitating tumor progression [190].

## 6 ROS as a Therapeutic Targets

Conventional therapeutic strategies, including chemotherapy and radiotherapy, exert their anti‐tumor effect through the generation of ROS. For instance, chemotherapeutic agents such as platinum‐based compounds and anthracyclines cause oxidative damage and subsequent activation of apoptotic signaling pathways by the accumulation of ROS. Likewise, the combined use of cisplatin and FK228 causes an increase in ROS levels, resulting in DNA damage and breast cancer cell death [191]. Radiotherapy is also a highly recognized method for the treatment of solid tumors. It essentially works by generating elevated ROS and free radicals through ionizing radiation. This leads to severe oxidative stress and can cause cellular DNA damage, ultimately promoting apoptotic cell death [192]. While traditional therapies are effective in targeting cancer cells, they often result in off‐target effects and are associated with an increased resistance to commercially available drugs, highlighting the need for more selective and modulated therapeutic strategies [193].

### 6.1 Nanomedicines

Conventional cancer therapies face the inherent challenge of restricted ROS generation due to the unfavorable conditions present in the TME, such as hypoxia, elevated ROS levels, and weakly acidic pH [194]. However, over recent years, several compelling studies have been conducted that elucidate the importance of nanomaterials in cancer therapeutics. By leveraging the stimuli‐responsive properties, nanomedicines hold immense potential to address the dynamic and complex conditions present in the TME, thereby allowing tailored and localized therapeutic responses [195]. These nanomaterials facilitate the targeted delivery and precise release of therapeutic payloads at specific sites upon encountering certain internal or external stimuli, potentially enhancing the therapeutic efficacy and minimizing off‐target effects [196]. For instance, in 2020, An et al. designed a pH‐sensitive ROS‐based drug delivery nanoplatform that effectively eliminated tumor cells by precise mitochondrial targeting. It can also be co‐loaded with anti‐cancerous drugs such as DOX and Ce6, the acidic TME providing favorable conditions for accelerated drug release. This treatment was used in combination with conventional strategies such as chemotherapy and sonodynamic therapy (SDT) [197]. Hypoxia or low oxygen concentration is a typical characteristic of the TME, which greatly impedes the anti‐cancerous effect of PDT. The MnO_2_@TPP‐PEG nano‐enzyme system was engineered to selectively modulate the catalysis of H_2_O_2_ in tumor cells to generate oxygen, enhancing the effect of PDT [198]. For the synergistic modulation of the TME, a pH/ROS responsive nanosystem was engineered that was loaded with siTGF‐β for chemoimmunotherapy. The charge‐reversal and size minimization potential of the nanosystem led to a profound antitumor immune response. The nanocarrier can escape from the endo/lysosomal degradation, followed by its disassembly and the subsequent release of the drug in elevated ROS levels [199]. Another synergistic approach utilizing oxidative therapy and chemotherapy demonstrated remarkable therapeutic effects by engineering a pH/ROS‐sensitive nanodrug that evokes a cascade of reactions within the TME, leading to the suppression of tumor growth [200].

Wang et al. specifically modulated the tumor microenvironment as a bioreactor for the biosynthesis of DNA molecules with metal precursors such as Au III and Fe II. The resultant self‐assembled Au&Fe_3_O_4_—DNA complex in situ served as a potent therapeutic agent for the precise and targeted imaging of tumor cells [201]. Moreover, it reinforces the photothermal effect with an increase in Au&Fe_3_O_4_—DNA complexes under near‐infrared radiation (NIR). It is also responsible for suppressing tumor growth by the intracellular depletion of glutathione (GSH) that leads to the apoptotic death of tumor cells. Thus, by dual regulating the levels of GSH and ROS in tumor cells, this TME‐responsive bioreactor serves as a favorable strategy for inhibiting tumor cells by intensifying oxidative stress in cancer treatment [201].

Wang et al. analyzed the anti‐tumor potency of Vanadium doped with Titanium (V‐TiO_2_) nano spindles. The fabricated nano spindles can act as Fenton‐like agents, catalyzing the conversion of increased levels of H_2_0_2_ in tumor cells to hydroxyl ions. Additionally, with the ability to deplete GSH, the oxidative stress generated by chemodynamic and sonodynamic therapies was intensified. In vitro and in vivo experiments further validated the oncolytic efficacy of V‐TiO_2_ nano spindles [202].

Additionally, a composite, multifunctional, and biodegradable nanoplatform for cancer therapy was fabricated by the deposition of CuS nanoparticles on surface‐functionalized MnO_2_ in a simple chemical co‐precipitation reaction followed by its further modification with hyaluronic acid (HA). The modified HA‐CuS/MnO_2_ nanosheets were selectively able to target tumor cells, followed by disassembly in the TME, and eventually increasing the production of ROS through a cascade of chemical reactions. This synergistic therapy exhibited noticeable anticancer potential, thus limiting cancer progression [203]. To enhance the imaging and therapeutic efficacy of tumor cells, Chu et al. fabricated a potential carbon dot nanozyme, Mn‐CD, using manganese and toluidine blue (TB) as the starting materials. Mn‐CDs exhibited several advantages, such as enhanced visualization of tumor cells using magnetic resonance (MRI) imaging by reacting to the specific conditions of the tumor microenvironment, such as acidic nature and GSH levels [204]. The manganese‐doped carbon dots were much more stable and effective under light and resulted in an increased level of ROS. Furthermore, in vivo and in vitro studies showcased the accumulation of Mn‐CDs at the tumor sites, resulting in enhanced permeability and retention effects.

In another study conducted by Li et al., researchers synthesized a TME‐sensitive nanoreactor by the encapsulation of Na_2_S_2_O_8_ nanocrystals with hollow tetra‐sulfide‐mesoporous silica (HTSMS); the complex is then enclosed with EG‐Fe (II) for the amplification of therapeutic efficacy [205]. The Na_2_S_2_O_8_ @HTSMSEF complex leads to the elicitation of a series of chemical reactions involving dual cycling of SO^4−^ and OH^−^ ions. Immunodeficient mice suffering from HepG2 liver carcinoma were intravenously administered Na_2_S_2_O_8_ @HTSMSEF. This resulted in suppressed tumor growth due to amplified oxidative stress and enhanced NIR‐II fluorescence imaging due to the accumulation of Na_2_S_2_O_8_ @HTSMSEF at tumor regions. This could be due to the significant increase in the penetration and retention effect of the nanocrystals [205]. Moreover, a newly developed silver molecule cluster, Ag5, demonstrated strong anti‐tumor activity by concurrently inhibiting glutathione and thioredoxin signaling pathways, diminishing its anti‐oxidant effect in cancer cells. It significantly reduces cell viability in various cancer cell lines while exhibiting minimal cytotoxic effects on normal, healthy cells. Ag5 remains effective in hypoxic conditions, although its activity is slightly reduced compared to normal oxygen levels. The decreased activity is attributed to HIF‐1 signaling and altered mitochondrial oxygen usage. Notably, Ag5 led to a significant improvement in therapeutic outcomes by synergistically enhancing radiation‐induced cell death, particularly under hypoxic conditions, making it a promising cancer‐selective agent [206].

Several nanomedicines have demonstrated significant potential in cancer immunotherapies by enabling targeted regulation of TME and immune cell activation. For instance, in a study, Liu et al. investigated the anti‐tumor potency of zinc protoporphyrin IX polypeptide micelle (ZnPP PM). This ROS‐inducing ZnPP PM significantly suppressed tumor cell progression by modulating macrophage polarization, enabling a shift from tumor‐supportive phenotype M2 to tumoricidal phenotype M1 in solid tumors. An increase in intracellular ROS levels and downregulation of STAT3 expression were observed in bone marrow‐derived TAMs. Furthermore, the administration of ZnPP/PIC in melanoma‐bearing mice resulted in the significant activation of NK cells and T‐lymphocytes, leading to significant tumor reduction [207].

Likewise, in another recent study, Zou et al. designed a novel TME‐responsive ZnPP@FQOS nanosystem to enhance the therapeutic efficacy of fibroblast‐rich and immunosuppressive tumors, such as pancreatic cancer, by the remodeling of cancer‐associated fibroblasts (CAFs) and amplification of ROS. The formulated hybrid system co‐delivers and releases hydrophilic quercetin (Que) and hydrophobic Zinc protoporphyrin (ZnPP) sequentially, under the influence of high pH and glutathione found in TME. The early release of Que facilitates the remodeling of CAFs. This remodeling leads to the amplification of endogenous/exogenous ROS by various pathways, subsequent tumor cell apoptosis, and downregulation of antioxidant marker HO‐1. In addition to its direct cytotoxic effects, ZnPP@FQOS also triggers systemic immunity and significantly improves the outcomes of anti‐PD‐L1 immunotherapy [208] (Table 1).

**TABLE 1:** Potential effect of nanomedicine on tumor microenvironment.

No.	Nanomedicines	Mechanism	Ref
1.	ROS‐based drug delivery nanoplatform	Suppression of tumor growth; can also be co‐loaded with anti‐cancerous drugs such as DOX and Ce6 (DOX release rate = 63.91 ± 1.67%)	[197]
2.	MnO_2_@TPP‐PEG	Tumor‐specific catalysis of H_2_O_2_ to enhance ROS‐mediated anti‐cancerous response	[198]
3.	pH/ROS responsive pro‐drug, loaded with siTGF‐β	Anti‐tumor immune response and enhanced the survival rate of tumor‐bearing mice by 75% within 35 days	[199]
4.	pH/ROS‐sensitive nanodrug	Accelerated drug release	[200]
5.	Au&Fe_3_O_4_—DNA complex	Intracellular depletion of Glutathione (GSH), inducing oxidative stress and consequently leading to apoptosis	[201]
6.	V‐TiO_2_ nano spindles	Acted as a Fenton‐like agent by enhancing the conversion of increased levels of H_2_0_2_ in tumor cells to hydroxyl ions	[202]
7.	HA‐CuS/MnO2 nanosheets	Noticeable anticancer potential by enhanced production of ROS	[203]
8.	Mn‐CDs	Increased levels of ROS, enhanced permeability, and retention effects	[204]
9.	Na_2_S_2_O_8_ @HTSMSEF complex	Suppressed tumor growth by amplified oxidative stress and enhanced NIR‐II fluorescence imaging	[205]
10.	Ag5	Inhibition of Anti‐oxidant pathways (glutathione and thioredoxin signaling pathways); highly effective anti‐tumor potency when combined with radiotherapy	[206]
11.	ZnPP PM	Suppression of tumor cell progression by macrophage polarization	[207]
12.	ZnPP@FQOS	Facilitates CAF remodeling, amplification of endogenous/exogenous ROS and inhibition of antioxidant system	[208]

### 6.2 Anti‐Oxidant Inhibiting Therapies

Glutathione (GSH) is considered an important anti‐oxidant to maintain ROS homeostasis in tumor cells. Elevated levels of GSH are observed in tumor cells because it helps to neutralize the production of ROS. GSH depletion has been reported to disrupt intracellular redox homeostasis, leading to an increase in ROS accumulation in tumor cells [209, 210]. If ROS levels increase beyond a certain threshold, it can lead to oxidative stress, ultimately causing cytotoxic effects. Therefore, there is a heightened interest in the development of strategies focusing on the indispensable role of GSH depletion in increasing the therapeutic efficacy of ROS‐based therapy. GSH depletion strategies are synergistically used with conventional cancer therapies to increase oncological outcomes. In a study conducted by Yoo et al., benzoyloxy dibenzyl carbonate, bc2, was synthesized as a pro‐oxidant, which can deplete GSH and elevate ROS levels, potentially inducing cell death [211]. It promoted mitochondrial disturbance, activation of PARP1, and cleavage of Bcl2, causing cancer cells to undergo apoptosis. Furthermore, the administration of bc2 in tumor‐bearing mice resulted in significant inhibition of tumor growth, with no adverse side effects detected. Correspondingly, another research demonstrates the use of small extracellular vesicles (sEVs) encapsulating B2C (BsEVs) as drug delivery agents, capable of circumventing the efflux system and delivering the drugs directly into the cytoplasm through endocytosis. It induces oxidative stress by GSH depletion in drug‐resistant (OVCAR‐8/MDR) and non‐resistant (OVCAR‐8) cancer cells, which subsequently leads to apoptotic cell death [212]. BsEVs were also shown to effectively enhance the drug sensitivity of cancer cells by inducing mitochondrial dysfunction. This disrupts the activity of the efflux pumps, which are used by the cancer cells in developing drug resistance.

Furthermore, coupling photodynamic therapy with a synthesized conjugate compound ZnP_C_‐C8‐Len, in which a VEGFR inhibitor (Lenvatinib) was linked to a photosensitizer (ZnP_C_), was used to reverse multi‐drug resistance (MDR) to enhance therapeutic outcomes. Upon irradiation, depletion of intracellular GSH levels led to the activation of the Bcl2/caspase 3 pathway ultimately inducing apoptotic cell death [213, 214]. Encapsulation of the nanoparticles with PEG_2000_‐PLA_2000_ caused a marked increase in tumor accumulation and antitumor activity. The fluorescence property of ZnPC‐C8‐Len assisted in the visualization of the tumor regions with greater effectiveness. Additionally, a chemotherapeutic drug Imexon displayed cytotoxic effects by binding to the thiol group of reduced GSH. Exposure to imexon led to GSH depletion, consequently inducing ER stress response and oxidative stress in pancreatic cancer cells [214] (Table 2).

**TABLE 2:** Summary of anti‐oxidant inhibiting therapies.

No.	Anti‐Oxidant Inhibiting Drugs	Mechanism	Ref
1	Benzoyloxy dibenzyl Carbonate, bc2	Depletion of GSH by inducing oxidative stress, Significant inhibition of tumor growth in tumor‐bearing mice	[211]
2	BsEVs	GSH depletion in drug‐resistant (OVCAR‐8/MDR) and non‐resistant (OVCAR‐8) cancer cells leads to apoptosis	[212]
3	Encapsulation of ZnP_C_ C8‐Len in PEG_2000_‐PLA_2000_	Depletion of intracellular GSH levels with increased anti‐tumor activity	[213]
4	Imexon	Increased oxidative stress in ER	[214]

### 6.3 Disrupting Mitochondrial Functioning

#### 6.3.1 Through Pro‐Oxidant Drugs

Several drugs that are approved for clinical use in the treatment of cancers are known to be potent inducers of oxidative stress, often targeting the cancer stem cell population (CSC). However, chemoresistance is a major challenge faced by chemotherapeutic drugs such as doxorubicin and anthracycline that need to be addressed duly to maximize therapeutic efficacy [215].

Elesclomol is a highly effective anti‐cancerous agent that can be used independently or in combination with other chemotherapeutic drugs such as paclitaxel to enhance its therapeutic efficacy against cancerous cells [216]. Additionally, numerous clinical trials are being conducted to analyze the anti‐cancerous potential of elesclomol in the treatment of prostate cancer, lung cancer, and soft tissue sarcoma [217]. It essentially acts in the presence of copper by targeting mitochondrial metabolism. It enhances the intracellular ROS levels and disrupts mitochondrial functioning, triggering cell death.

Another pro‐oxidant drug Celecoxib (CXB) demonstrated cytotoxic activity against metastatic breast cancer cells even at low concentrations. It selectively disrupted the mitochondrial transmembrane potential, ultimately causing ROS‐mediated apoptosis in metastatic cancer cells [218]. Likewise, in another study, CXB targeted the VEGF promoter, which resulted in the inhibition of VEGF expression, thereby inhibiting angiogenesis and potentially slowing down tumor growth and progression [219].

Furthermore, the role of Cabazitaxel (Cab) in the treatment of prostate cancer is well established. However, it is usually combined with other immunotherapies or targeted treatments to increase its effectiveness. Recently, Eryilmaz and colleagues investigated the pro‐oxidant activity of Cab on prostate cancer cell lines. Results revealed that Cab was able to induce ROS‐mediated apoptotic cell death. Conversely, it also enhanced the expression of p‐NF‐κB in cancer cells, which potentially hints at a mechanism used by cancer cells to evade apoptotic death in the presence of increased intracellular ROS levels. Further research is thus required to unveil the intricate role of Cab [220].

Auranofin is a clinically approved drug for rheumatoid arthritis; however, recently, it has garnered immense attention as a potent pro‐oxidant drug. It is well known to trigger intracellular oxidative stress in different cancer cell lines and tumor models. It selectively targets and inhibits the activity of the thioredoxin reductase (TrxR) pathway, which is an anti‐oxidant system and protects the cell from oxidative damage. This results in the disruption of normal redox homeostasis and results in increased intracellular ROS levels, followed by the stimulation of cell death [221] (Table 3).

**TABLE 3:** Disruption of mitochondrial function by synthetic pro‐oxidant drugs.

No.	Pro‐oxidant Drugs	Mechanism	Ref
1.	Elesclomol	Functions in the presence of copper by targeting mitochondrial metabolism	[215, 216]
2.	Celecoxib (CXB)	Stimulation of ROS‐mediated apoptosis, inhibition of VEGF expression and angiogenesis	[217, 218]
3.	Cabazitaxel (Cab)	Triggers ROS‐mediated apoptotic cell death	[219]
4.	Auranofin	Stimulation of intracellular oxidative stress in different cancer cell lines and tumor models and inhibition of the thioredoxin reductase (TrxR) pathway	[220]

#### 6.3.2 Through Phytochemicals

The synergistic or independent role of certain natural compounds present in plants in selectively modulating the cancer hallmarks, such as inhibition of cell cycle proteins, disrupting angiogenesis, suppressing migration invasion, and stimulating apoptosis has sparked great interest among researchers [222, 223].

The anti‐cancerous role of Neferine (Nef), an alkaloid present in *Nelumbo nucifera* , is evident from a study conducted by Dasari et al. where Nef considerably reduced the viability of cancer cell lines (HeLa and SiHa) while displaying minimal toxicity to normal cells. It had the potential to suppress the metastatic growth of cancer cells. Moreover, it enhanced the expression of apoptotic and autophagic proteins, thus stimulating both cell death regulatory pathways through ROS involvement [224]. Likewise, Resveratrol demonstrated an inhibitory effect on the Nrf2 signaling pathway in several cancer cells [225]. It induced the apoptotic pathways in a dose‐and time‐dependent manner by increasing ROS levels, thus suppressing tumor cell progression [226]. Additionally, it modulated the tumor microenvironment by promoting the inhibition of HIF‐1 generation, which in turn prevented the secretion of VEFG, thus impacting angiogenesis. It also had the potential to suppress fibrosis‐promoting agents such as fibronectin and type I collagen [227].

Thymoquinone (TQ) has exhibited the ability to inhibit the proliferative activity of different cancer cells through ROS‐driven inhibition of the STAT3 pathway, thereby inducing apoptosis in cancer cells. TQ was shown to upregulate the expression of p53, Bax proteins, and caspases. Moreover, the expression of STAT3 target genes, cyclin D1, and survivin was greatly reduced by the activity of TQ. In vivo studies further validated the role of TQ in stimulating apoptosis of A431 cancer cells by enhancing ROS production and disrupting the STAT3 signaling pathway [228]. Similarly, another study reported the role of TQ in inducing oxidative stress and increasing ROS generation, thereby demonstrating inhibitory effects on A549 lung cancer cells [229].

A study highlighted the role of Silibinin, an important component of *Silybum marianum* , in activating cancer cell apoptosis and enhancing ROS levels by the stimulation of the JNK/c‐JUN pathway [230]. JNK pathway activation leads to mitochondrial dysfunction via Bax protein activation and Bcl‐2/Bcl‐xl inhibition, thereby inducing apoptosis. Moreover, the expression of p53 was significantly increased in cancer cells treated with silibinin. Yet another study demonstrated the promising role of silibinin against breast cancer cells when combined with chemotherapeutic drugs such as doxorubicin, cisplatin, and carboplatin [231].

Another important plant constituent, Evodiamine, is currently being explored for its anti‐cancerous potential by modulating different signaling pathways. It can restore drug responsiveness in different cancer cells [232]. For instance, in a study, EVO was shown to regulate the apoptotic pathway and survival signal transduction pathways to effectively increase doxorubicin sensitivity in doxorubicin‐resistant cancer cells [233]. The stimulation of apoptosis occurred through a caspase‐dependent pathway. EVO also suppressed the Ras/MEK/ERK pathway and inhibitors of apoptosis (IAPs) (Table 4).

**TABLE 4:** Disruption of mitochondrial function by natural phytochemicals.

No.	Phytochemicals	Mechanism	Ref
1.	Neferine (Nef)	Stimulation of cell death regulatory pathways by upregulating the expression of apoptotic and autophagic proteins	[223]
2.	Resveratrol	Inhibitory effect on the Nrf2 signaling pathway, activation of apoptotic cell signaling pathway and suppression of fibrosis‐promoting agents such as fibronectin and type I collagen	[224, 225, 226]
3.	Thymoquinone (TQ)	Upregulation of p53, Bax proteins, and caspases and stimulation of apoptosis of A431 cancer cells by enhancing ROS production	[227, 228]
4.	Silibinin	Activation of cancer cell apoptosis and enhanced ROS levels by the stimulation of the JNK/c‐JUN pathway, anti‐cancerous activity against breast cancer when combined with chemotherapeutic drugs	[229, 230]
5.	Evodiamine	Restore drug responsiveness in different cancer cells, stimulation of apoptosis and suppression of the Ras/MEK/ERK pathway	[231, 232]

## 7 Conclusion and Future Perspectives

This review has elucidated the intricate role of ROS in tumor progression and dissemination. The perplexing relation between ROS and cancer is based on the precise regulation of ROS homeostasis. Understanding the multifaceted role of ROS in TME is extremely important during various stages of cancer development. ROS plays a critical role in TME by influencing various aspects of tumor progression and the host immune response. Conventional strategies to manage tumor progression and cancer might not be effective in the long run due to the development of ROS‐mediated drug resistance and stimulation of systemic toxicity [234]. ROS modulation strategies can, however, prove to be a promising approach in cancer therapeutics as tumor cells substantially differentiate themselves from their normal counterparts by exhibiting altered ROS levels. Nanomedicine holds great potential in cancer treatment by acting as an effective drug delivery platform with stimuli‐responsive drug release. These ROS‐responsive platforms are usually used synergistically with conventional treatments such as chemotherapy or sonodynamic therapies, leading to enhanced therapeutic outcomes [197, 198, 199, 200, 201, 202, 203, 204, 205]. However, translating ROS‐based therapeutic strategies, such as nanomedicines, from bench to bedside is a complex and multilayered process. One of the significant challenges is precisely adjusting the responsiveness of the nanosystems to selectively respond to the elevated ROS levels in the tumor microenvironment [235]. Another factor that represents a significant constraint is the maintenance of batch‐to‐batch consistency, as even minor changes can impact therapeutic and safety efficacy [236]. To enhance the clinical precision of nanomedicines, certain parameters such as the risk assessment, toxicology profiles, and potential long‐term health effects of nanomaterials should be addressed by developing safety and regulatory frameworks [234]. Moreover, the development of GSH‐depleting agents by suppressing antioxidant systems is another effective therapeutic approach that is used in combination with traditional therapies. GSH‐depleting agents disrupt the normal redox balance, resulting in elevated ROS levels and depleted GSH levels that potentially target tumor cells [211, 212, 213, 214]. Careful consideration of selective targeting, clinical effectiveness, and side effects is essential for the effective implementation of this strategy in cancer therapeutics. Certain pro‐oxidant drugs and phytochemicals are also reported to modulate the TME by regulating different signaling pathways involved in cancer development and progression [215, 216, 217, 218, 219, 220, 221, 222, 223, 224, 225, 226, 227, 228, 229, 230, 231, 232]. Despite noticeable potential in preclinical studies, the clinical translation of ROS‐modulating therapies has generated mixed results. For instance, ATN‐224 has demonstrated substantial anti‐angiogenic activity and can suppress cell proliferation while inducing cell death in various cancer cell lines. However, when tested in phase II clinical trials for prostate cancer, ATN‐224 failed to show a clear dose–response therapeutic response. Moreover, the specific signaling pathway through which ATN‐224 exerts its antitumor effects in vitro is still unclear, further restricting its clinical potential as an anti‐cancer agent, either alone or in combination with other therapies [237]. Similarly, the TRX1 inhibitor PX‐12 showed noticeable anti‐tumor activity in preclinical studies and early‐phase clinical trials. However, the anticipated therapeutic efficacy was not observed in phase II clinical trials, thereby limiting its clinical advancement [238, 239]. Correspondingly, Phase I and II clinical trials demonstrated the substantial therapeutic potential of NOV‐002, a GSSG analog, when administered in combination with carboplatin/paclitaxel, but it did not yield a significant improvement in overall survival of patients in advanced non‐small cell lung cancer in Phase III trials [240]. Hence, further research and clinical studies are required to validate the use of these agents in effective cancer management.

## Author Contributions

I.A. and Z.H. contributed equally to the manuscript and share first authorship. I.A., Z.H., and M.K. were responsible for conceptualizing the review structure, gathering literature, and drafting the initial manuscript. S.H. contributed to the writing and critically revised the manuscript for biological accuracy. M.M.S. provided expertise on oxidative stress pathways and therapeutic interventions and reviewed and edited the manuscript for scientific rigor. W.N.I. supervised the project, contributed to the conceptual framework, critically revised the content related to tumorigenesis and therapy strategies, and finalized the manuscript for submission. S.A. conceptualized and coordinated the project, contributed to manuscript writing, and provided overall supervision. All authors read and approved the final manuscript.

## Conflicts of Interest

The authors declare no conflicts of interest.

## Data Availability

The data that supports the findings of this study are available in the supporting information of this article.
